# Effect of Time of Day of Infection on Chlamydia Infectivity and Pathogenesis

**DOI:** 10.1038/s41598-019-47878-y

**Published:** 2019-08-06

**Authors:** Stephanie R. Lundy, Tarek Ahmad, Tankya Simoneaux, Ifeyinwa Benyeogor, YeMaya Robinson, Zenas George, Debra Ellerson, Ward Kirlin, Tolulope Omosun, Francis O. Eko, Carolyn M. Black, Uriel Blas-Machado, Jason P. DeBruyne, Joseph U. Igietseme, Qing He, Yusuf O. Omosun

**Affiliations:** 1 Department of Microbiology, Biochemistry & Immunology, Morehouse School of Medicine, Atlanta, GA 30310 USA; 2 Molecular Pathogenesis Laboratory, Centers for Disease Control & Prevention (CDC), Atlanta, GA 30333 USA; 3 Department of Pharmacology and Toxicology, Morehouse School of Medicine, Atlanta, GA 30310 USA; 4 Department of Physical Sciences, Georgia State University, Covington, GA 30014 USA; 5 Department of Pathology and Athens Veterinary Diagnostic Laboratory, University of Georgia, College of Veterinary Medicine, Athens, GA 30602 USA; 6 Spelman College, Atlanta, GA 30314 USA

**Keywords:** Bacterial host response, Bacterial pathogenesis

## Abstract

Genital chlamydia infection in women causes complications such as pelvic inflammatory disease and tubal factor infertility, but it is unclear why some women are more susceptible than others. Possible factors, such as time of day of chlamydia infection on chlamydial pathogenesis has not been determined. We hypothesised that infections during the day, will cause increased complications compared to infections at night. Mice placed under normal 12:12 light: dark (LD) cycle were infected intravaginally with *Chlamydia muridarum* either at zeitgeber time 3, ZT3 and ZT15. Infectivity was monitored by periodic vaginal swabs and chlamydiae isolation. Blood and vaginal washes were collected for host immunologic response assessments. The reproductive tracts of the mice were examined histopathologically, and fertility was determined by embryo enumeration after mating. Mice infected at ZT3 shed significantly more *C. muridarum* than mice infected at ZT15. This correlated with the increased genital tract pathology observed in mice infected at ZT3. Mice infected at ZT3 were less fertile than mice infected at ZT15. The results suggest that the time of day of infection influences chlamydial pathogenesis, it indicates a possible association between complications from chlamydia infection and host circadian clock, which may lead to a better understanding of chlamydial pathogenesis.

## Introduction

Genital chlamydia infection, a sexually transmitted infection (STI), which is caused by the bacteria C. trachomatis, is the most commonly reported bacterial STI in the United States. In the female reproductive tract, genital chlamydia infection is manifested in several ways such as pelvic inflammatory disease (PID), Salpingitis and tubal factor infertility^1–3^. A high number of chlamydia infection cases go unreported because they are asymptomatic, and this has been estimated to be about 70% of the cases in women^2,3^. There are varying levels of severity among women who do develop complications such as cervicitis, PID, uterine fibrosis and tubal factor infertility (TFI) regardless of how many times they have been exposed or have become reinfected^4,5^. A better understanding of how some women develop genital disease (PID alone, PID with Salpingitis, TFI or are asymptomatic) following chlamydia infection, while others do not, and the underlying mechanisms^6,7^ will enhance a search for prevention measures.

Circadian rhythms, the body’s internal clock, describe the endogenous oscillation in organisms that are observed in approximate association with the Earth’s daily rotation^8^. Circadian rhythms are governed by the light-dark cycle and allows organisms to anticipate changes in the environment^8^. In mammals, circadian rhythms are regulated by the molecular clock of the suprachiasmatic nucleus (SCN) of the hypothalamus and by circadian clocks found in most peripheral tissues^9^. The molecular clock consists of several transcription factors that function in an autoregulatory transcription-translation feedback loop^8^. The circadian genes, *Clock* and *Bmal1*, induce the transcription of clock genes *Period 1* and *Period 2* (PER) and *Cryptochrome 1* (CRY) and *Rev-erb*^8^. Following translation, PER and CRY proteins dimerize and re-enter the nucleus to inhibit their own transcription acting as a negative feedback loop^8,10,11^. This core circadian feedback loop drives ~24-hour rhythms in the expression of other ‘output’ genes, ultimately leading to overt rhythms in behaviour and physiology. In addition, there are additional circuits that cooperate with the core clock. This involves, RAR-related orphan receptor, RORα, that has similar DNA binding sites with REV-ERBs, it induces expression of *Bmal1* in a feed forward loop, whereas REV-ERBs repress transcription of *Bmal1* by competing for the same site in a negative feedback loop. Furthermore, albumin D-box binding protein (DBP) and the repressor nuclear factor interleukin 3 (NFIL3 or E4BP4) form an extra loop, which regulates transcription of genes containing D-box, sequences, including Period. These rhythmic feedback mechanisms generate oscillations in gene expression that convey circadian timing cues to cellular processes^12^.

Circadian rhythms influence several physiological processes in the body including immune response and reproduction as well as influence antibacterial host defences, sepsis, inflammation and cell proliferation^8,13–15^. Understanding the role of circadian rhythms on bacterial infections has been of interest for a long time^16,17^. Other studies conducted on pathogens such as human herpes virus 2, influenza, and *Salmonella typhimurium* suggests the outcomes of these infections are regulated by circadian clocks in the infected organisms^18,19^. It is unclear if circadian clocks influence the pathogenesis of genital chlamydia infection. Genital chlamydia infection is controlled by the immune system, starting from the epithelial cells at the site of infection, which are the first to secrete proinflammatory cytokines/chemokines such as C-X-C Motif Chemokine Ligand 1 (CXCL1), IL-8, CXCL16, IL-6 and tumor necrosis factor (TNF)^20–22^. Immune cells respond to the chemokines produced by the infected epithelial cells and are recruited to the site of infection. T cells especially the CD4+ T cells play an important role in clearing chlamydia through a Th1 dependent response, by secreting IFN-γ and IL-1β that help in clearing and reducing the bacterial burden^23–26^. In addition, toll-like receptors, which recognize microbial antigens, play an important role in the immune response to chlamydia. They help in stimulating dendritic cells, which in turn produce IL-12 that induces Th1 type response^27,28^. The innate and adaptive immune system in mice and human have been shown to be regulated by endogenous circadian clocks^29^. They have been reported to display daily rhythms of cell counts in blood and peripheral lymphoid organs, lymphocyte proliferation, and *in vitro* stimulated cytokine levels^29–44^. For instance, a conserved circadian clock has also been reported in peripheral blood mononuclear cells (PBMCs), and the phase of gene expression in these immune cells have been shown to be sensitive to light and dark^39,45,46^. Immune cells such as neutrophils, macrophages, dendritic cells, T and B cells have all been shown to be under the control of intrinsic circadian clocks^29,34^, which appears to be associated with rhythmic functions such as cytokine and antibody production. However, the number of circulating immune cells seems to be under the control of the central clock through humoral and neuronal signalling^33,47–49^. Studies focused on *Bmal1*^−/−^ and infertility have shown that female *Bmal1*^−/−^ mice are either infertile or their fertilized egg could not successfully implant^14^. This suggests that *Bmal1* plays an important role in reproduction^14^.

Altogether, circadian clocks have been shown to regulate some viral and bacterial infections^18,19^. However, we do not know the role of host circadian rhythms on the pathogenesis of genital chlamydia infection. We hypothesized that genital chlamydia infection is regulated by circadian clocks present in the infected organisms, however this study was not designed to discern between circadian rhythms directed by the SCN or the oscillations of immune modulators directed by the circadian clocks in the immune cells. The current study was designed to determine the influence of time of day on chlamydia infection and complications, to help us better understand the processes involved in chlamydia pathogenesis that leads to infertility.

## Results

### Effect of Time of Day on *C. muridarum* infectivity

**Figure 1:**
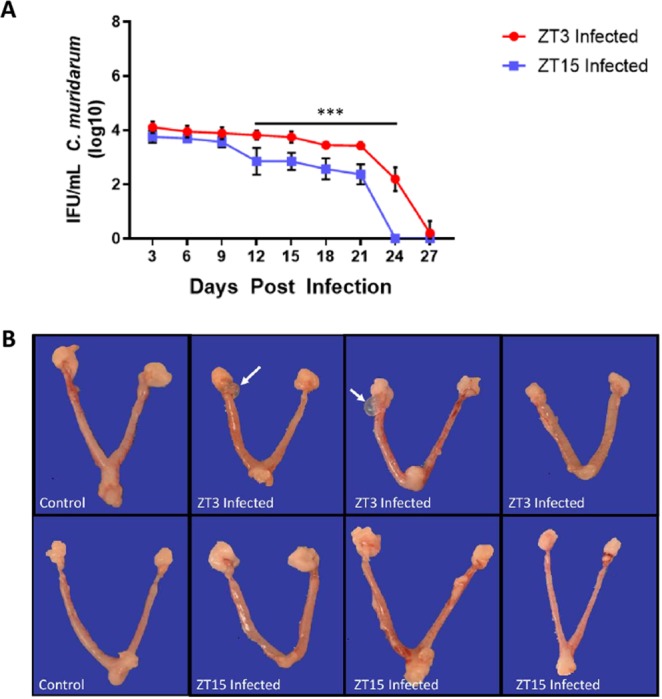
Chlamydia Infectivity and gross pathology in female mice housed under LD conditions. Mice (n = 12 per group) were infected with *C. muridarum* at either ZT3 or ZT15. Experiment was repeated twice. (**A**) Mice infected at ZT3 had a significantly higher bacterial burden compared to mice infected at ZT15 from days 12 and 24 (***p < 0.001). Data was analysed using two-way analysis of variance (ANOVA). (**B**) Gross pathology in female mice housed under LD conditions. Note, mice infected ZT3 had paraovarian (next to the ovary) cysts as indicated by the white arrow, mice infected at ZT15 did not have paraovarian cysts.

Female mice housed under 12:12 light: dark (12:12LD) were infected with *C. muridarum* at either 3-hours after lights-on, Zeitgeber Time 3, (ZT3), or 3-hours after lights-off (ZT15). These time-points were selected because they coincide with the early rest period (ZT3) and early active period (ZT15) of the nocturnal mice. When the infectivity was measured by measuring shedding of chlamydiae into the cervico-vaginal vault, mice infected at ZT3 had significantly higher infectivity compared to the mice infected at ZT15 from days 12–24 post-infection (p < 0.0001) (Fig. 1A). These results suggest that mice infected at ZT15 had lower infectivity from days 12–24 and had cleared the infection by day 24 while mice infected during the day ZT3 had higher infectivity from days 12–24 and did not clear the infection until day 27. The results imply that the time of day in which mice are exposed to pathogen plays an important role in *C. muridarum* infectivity.

### Effect of Time Day of *C. muridarum* infection on the female reproductive tract pathology

**Figure 2:**
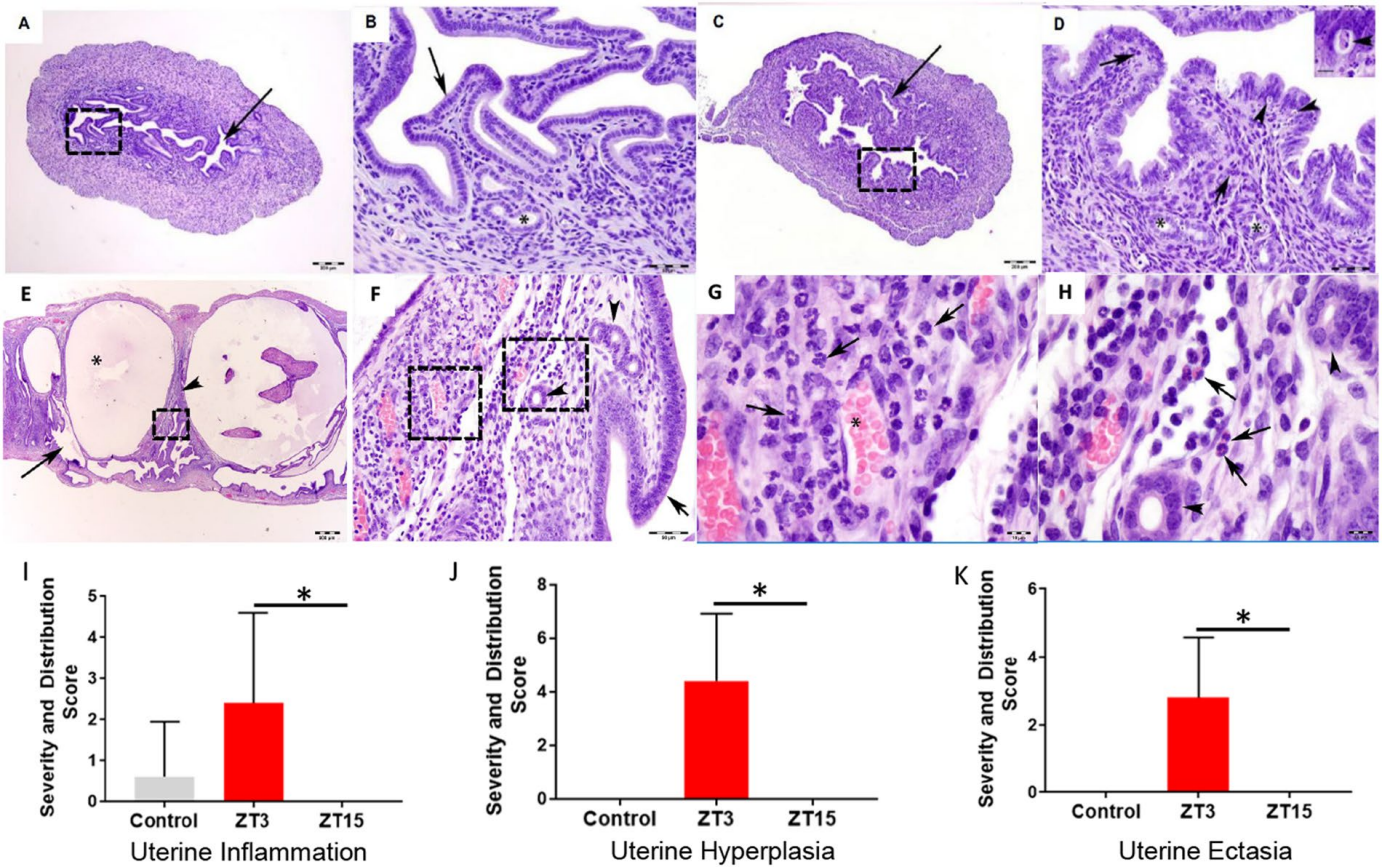
Histopathology in female mice housed under LD conditions. Mice (n = 12 per group) were infected with *C. muridarum* at either ZT3 or ZT15. (**A)** Representative image of the uterus from a mouse infected at ZT15. Arrow points to the normal endometrium. H and E stain. Scale bar = 200 μm. (**B)** Higher magnification of 2A (dashed rectangle). A layer of simple columnar epithelia lines the endometrium (arrow). Endometrial glands (asterisk, *) are surrounded by few stromal cells. H and E stain. Scale bar = 50 μm. (**C)** Representative image of the uterus from a mouse infected at ZT3. Arrow points to the hyperplastic and inflamed endometrium. H and E stain. Scale bar = 200 μm. (**D)** Higher magnification of 2C (dashed rectangle). A hypercellular (hyperplastic) layer of tall columnar epithelia with widely scattered apoptotic necrosis (arrowheads and inset) lines the endometrium. Within the lamina propria, there are increased numbers of eosinophils (arrows). Endometrial glands (asterisks, *). H and E stain. Scale bar = 50 μm; inset scale bar = 10 μm. (**E)** Representative image of the uterus with cystic endometrial hyperplasia and endometritis from a mouse infected at ZT3. Arrow points to the endometrium. Asterisk (*) is in the lumen of a cystic endometrial gland. Arrowhead points to the lamina propria. H and E stain. Scale bar = 500 μm. (**F)** Higher magnification of the lamina propria of 2E (dashed rectangle). A layer of columnar epithelia lines the endometrium (arrow). Few stromal cells and a mixture of eosinophils and neutrophils surround endometrial glands (arrowhead). Asterisk (*) is in the lumen of a cystic endometrial gland. H and E stain. Scale bar = 50 μm. (**G)** Higher magnification of the lamina propria of 2F (dashed rectangle on the left). Increased numbers of neutrophils (arrows) expand the lamina propria. Asterisk (*) is in the lumen of a blood vessel filled with erythrocytes. H and E stain. Scale bar = 10 μm. H**)** Higher magnification of 2F (dashed rectangle on the right). Increased numbers of eosinophils (arrows) expand the lamina propria. Individual eosinophils have an orange cytoplasm and a bilobed nucleus. Endometrial glands (arrowheads). H and E stain. Scale bar = 10 μm. The scores for the genital tract histopathology described were presented as follows, (**H)** Uterine inflammation. (**I)** Uterine hyperplasia. (**J)** Uterine ectasia. The histopathology scores were analysed using a one-way ANOVA and post hoc test.

Female mice housed under LD conditions were infected with *C. muridarum* at either ZT3 or ZT15. The incidence and severity of gross lesions on reproductive tract were more common in mice infected at ZT3 than those infected at ZT15 (Fig. 1B). The lesions consisted of increased numbers of uterine dilations and paraovarian cysts. Histopathologically, mice infected at ZT3 had evidence of increased incidence and severity of uterine inflammation, hyperplasia, and apoptotic necrosis when compared to mice that were infected at ZT15 (Fig. 2A–K). Mice infected at ZT15, however, experienced some uterine dilations but no histopathological evidence of significant genital tract pathology. These results indicate that the increase in infectivity observed in the mice infected during the early rest period has a positive correlation to the lesions observed in them.

### Effect of Time of Day of *C. muridarum* infection on cytokine production

**Figure 3:**
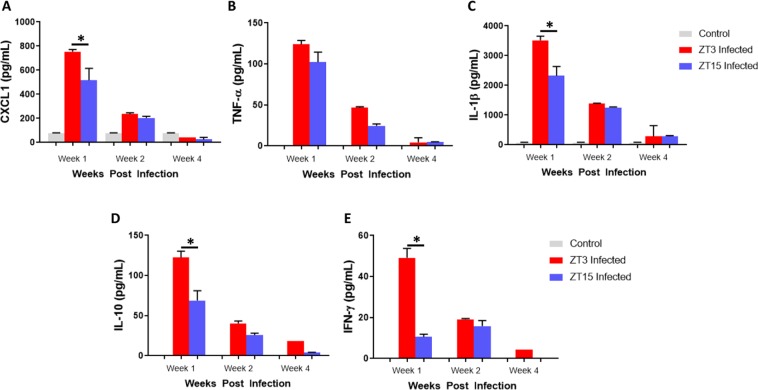
Chemokine/Cytokine determination in mice housed under LD conditions. Mice (n = 12 per group) were infected with *C. muridarum* at either ZT3 or ZT15 and serum samples were collected at different time points after infection. (**A**) Mice infected at ZT3 expressed significantly higher level of CXCL1 during the first week of infection compared to mice infected at ZT15 (**p < 0.01). (**B**) TNF-α was highly expressed in first week of infection in mice infected at ZT3 and mice infected at ZT15. However, there was no significant difference in expression levels. (**C)** Mice infected at ZT3 expressed significantly higher level of IL-1β during the first week of infection compared to mice infected at ZT15 (**p < 0.01). (**D**) Mice infected at ZT3 expressed significantly higher level of IL-10 during the first week of infection compared to mice infected at ZT15 (**p < 0.01). (**E)** Mice infected at ZT3 expressed significantly higher level of IFN-γ during the first week of infection compared to mice infected at ZT15 (**p < 0.01). The data was analysed using a one-way ANOVA and post hoc test.

We determined the influence of time of day of chlamydia infection on the immune response to *C. muridarum* by analysing the cytokines, chemokines and antibodies in vaginal lavages and serum samples. Under LD conditions, mice infected during the day had significantly higher levels of CXCL-1 during the first week of infection compared to mice infected at ZT15 (p < 0.01) (Fig. 3A). During the first and second weeks of infection TNF-α expression was higher in mice infected at ZT3 compared to mice infected at ZT15; however, this difference was not significant (Fig. 3B). Interleukin-1 β (IL-1β), Interleukin-10 (IL-10) and Interferon- γ (IFN-γ) were significantly higher in the first week of infection in mice infected at ZT3 compared to mice infected at ZT15 (p < 0.01, p < 0.01, p < 0.01 respectively) (Fig. 3C–E). The results reveal that there was a greater host inflammatory response to *C. muridarum* when infection took place during the early rest period than during the early active period. The observation corroborates the hypothesis that the host’s physiologic state at the time of infection, which might be controlled by circadian clocks, could influence the intensity and pathologic outcomes of genital chlamydia infection.

### Effect of Time of Day of *C. muridarum* infection on anti-chlamydial antibody production

**Figure 4:**
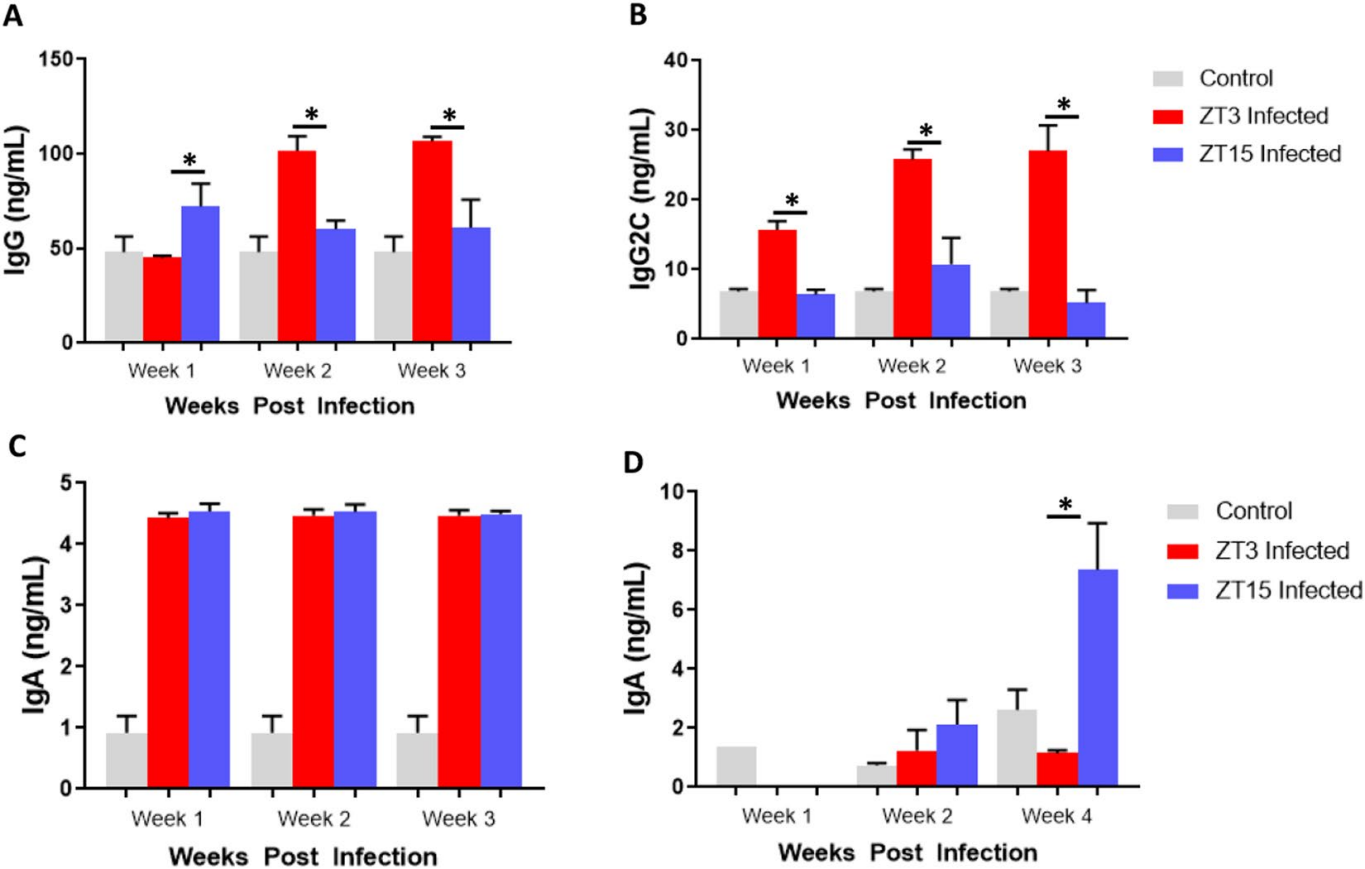
Anti-chlamydia antibody determination in mice housed under LD conditions. Mice were infected with *C. muridarum* at either ZT3 or ZT15 and serum samples were collected at different time points after infection. (**A**) IgG was significantly higher in mice infected at ZT15 in the first week of infection compared to mice infected at ZT3 (*p < 0.05). However, during weeks two and three of infection, IgG was significantly higher in mice infected at ZT3 compared to mice infected at ZT15 (**p < 0.01). (**B**) IgG2C was significantly higher in mice infected at ZT3 compared to mice infected at ZT15 (**p < 0.01, ****p < 0.0001). (**C**) No significant difference in IgA levels between mice infected at ZT3 or ZT15. (**D**) Significant increase in IgA levels in mice infected at ZT15 in the fourth week of infection compared to mice infected at ZT3 (**p < 0.01). The data was analysed using a one-way ANOVA and post hoc test.

Under LD conditions, in the first week of infection, chlamydia specific Immunoglobulin (Ig) IgG expression was higher in mice infected at ZT15 compared mice infected at ZT3 (p < 0.01). While, in second and third weeks of infection, chlamydia specific IgG secretion was significantly higher in mice infected at ZT3 compared to mice infected at ZT15 (p < 0.001) (Fig. 4A). In the first, second and third weeks of infection, there was a significantly higher secretion of chlamydia specific IgG2C in mice infected at ZT3 compared to mice infected at ZT15 (p < 0.001 and p < 0.01) (Fig. 4B). There was no significant difference in the secretion of serum chlamydia specific IgA at ZT3 and ZT15 infected mice (p > 0.05) (Fig. 4C). However, there was a noticeable difference in IgA secretion in the vaginal wash samples, with a significant increase by the fourth week of infection in mice infected at ZT 15 (p < 0.01) (Fig. 4D). The results show that total IgG in serum is important in mice infected during the early active period in the first week of infection. However, IgG2C seems more relevant in mice infected during the early rest period. While mucosal IgA in mice infected during the early active can be associated with chlamydia clearance.

### Effect of Time of Day on *C. muridarum* reinfection

**Figure 5:**
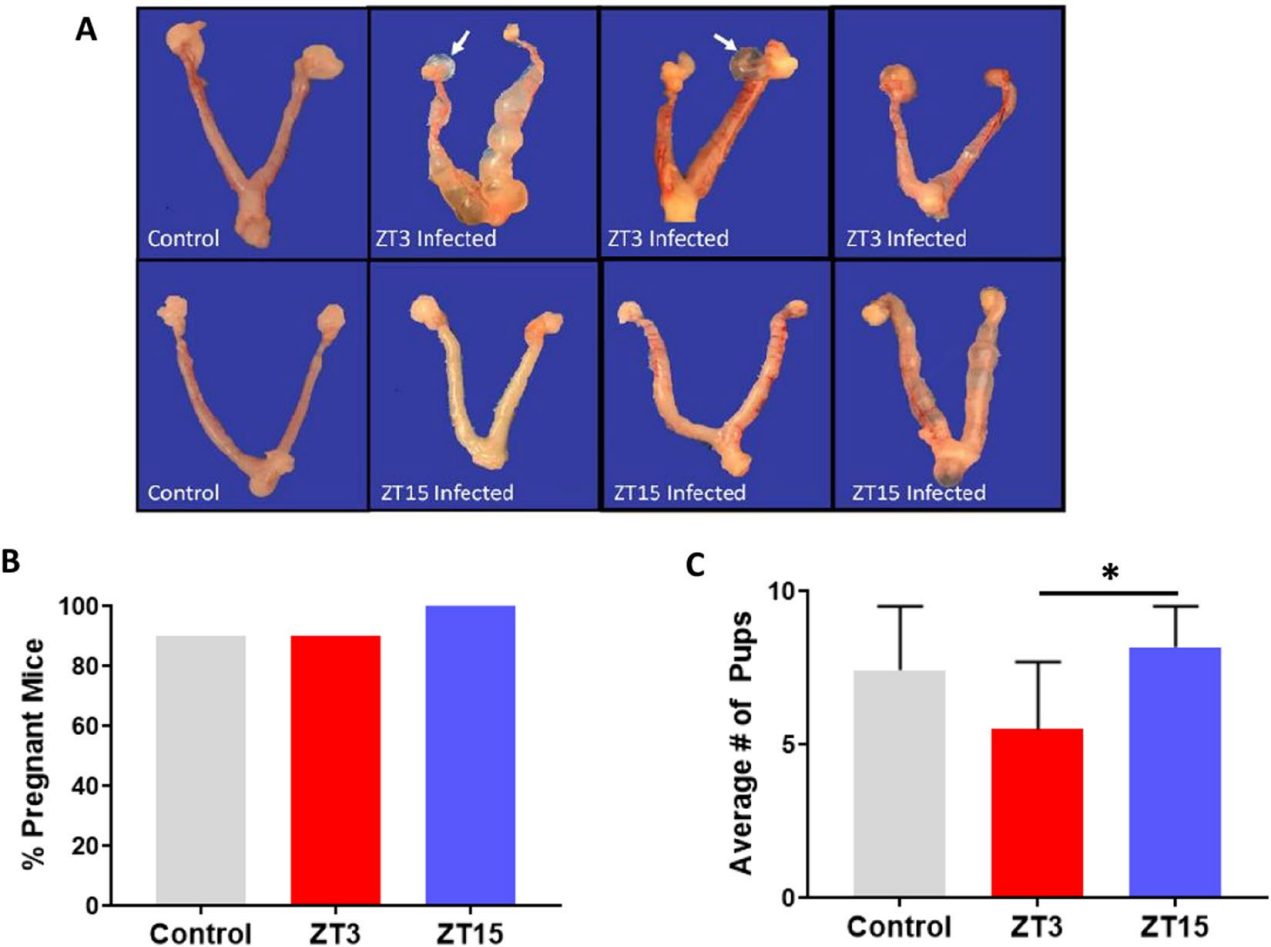
Effect of time of day of infection on reinfection under LD conditions. Mice (n = 18 per group) were infected with *C. muridarum* at either ZT3 or ZT15 and then reinfected at the same time of the day 4 weeks later. Experiment was repeated twice. (**A)** Mice infected at ZT3 had more uterine tubal dilations compared to mice infected at ZT15. Mice infected at ZT3 had paraovarian cysts while mice infected at ZT15 did not have paraovarian cysts. (**B)** There was no difference in fertility rate between the mice infected at ZT3 and ZT15, using Chi-square test. (**C**) Mice infected at ZT15 had a significantly greater number of pups compared to mice infected at ZT3 (*p < 0.05). The data was analysed using a one-way ANOVA and post hoc test.

In humans, complications due to genital chlamydia infection often occurs after repeat or untreated infections. Due to the differences observed in infectivity, pathology, and immune response associated with time of day of chlamydia infection, we wanted to determine if these differences would also be observed in mice with repeat infections. Female mice housed under LD conditions were reinfected with *C. muridarum* at either ZT3 or ZT15, to determine if repeat infection at the same time as the first infection had worse outcomes. The difference in bacterial burden following reinfection, between ZT3 and ZT15 infection was comparable to what we observed in a single infection. However, mice reinfected at ZT3 had more gross lesions compared to mice reinfected at ZT15, and only mice reinfected at ZT3 had paraovarian cysts (Fig. 5A) with half of the mice reinfected at ZT3 having uterine tubal dilations or hydrometra. There were no observable differences in uterine histopathology when comparing mice infected at either ZT3 or ZT15 with both groups presenting mild endometrial hyperplasia (increased numbers of cell in the endometrial lining), endometrial apoptotic necrosis (cell death) and hydrometra (accumulation of fluid in the cavity of the uterus). One mouse with hydrosalpinx associated with dilated oviductal ampulla and two mice with Bursal cysts were observed in the mice reinfected during the early rest period, and in this group had similar incidence of inflammation in their ovary compared to mice reinfected during the early rest period. An interesting observation was that between the ZT3 and ZT15 uninfected control groups, the incidence of endometrial hyperplasia was higher in the ZT3 group than in the ZT15 group. In contrast, the incidence of endometrial apoptotic necrosis was higher in the ZT15 control group than in the ZT3 control group. Following reinfection, control, ZT3 and ZT15 reinfected mice were mated to determine the effect of time of day of infection on pregnancy and fertility rate. There was no significant difference in the pregnancy rate between the ZT3 and ZT15 reinfected mice (Fig. 5B), however, mice infected during the early active period had a significantly greater number of pups compared to mice infected during the early rest period. Mice reinfected during the early active period had an average of eight pups while mice infected during the early rest period had an average of 5 (p < 0.05) (Fig. 5C). The results suggested that the effect of time of day on chlamydial pathogenesis is the same during a repeat infection, furthermore time of day of infection influences the overall fertility rate of the infected mice.

### Effect of time of day of infection on LGV pathogenesis

We extended the observed effect of time of day of infection on the infectivity and pathogenesis of the mouse chlamydia strain, *C. muridarum*, to the human *C. trachomatis* strains to rule out any unique feature of the mouse strain and establish that time of day of infection affects all chlamydia species. Female mice housed under LD conditions were infected with the *C. trachomatis* agent of *lymphogranuloma venereum (*LGV) at either ZT3 or ZT15. Like the mice infected with *C. muridarum*, mice infected with LGV at ZT3 had significantly higher infectivity compared to mice infected at ZT15 from days 19 and 26 (p < 0.0001) (S. Fig. 1). Mice infected at ZT15 cleared the infection by day 26 while mice infected at ZT3 did not fully clear their infection even at 26 days post infection. On histopathology, however, there were no significant differences in the incidence and severity of lesions observed between control mice and mice infected during early active or early rest periods. Overall the uterus of mice infected during the early rest period and mice infected during the early active period were not significantly different histopathologically, however the ovaries of mice infected during the early rest period had more inflammation (S. Fig. 2). Following reinfection, we observed a trend in fertility in *LGV* infected mice like *C. muridarum* infected mice. Mice infected during the early rest period had a lower pregnancy rate than mice infected during the early active period and they had significantly less pups (p < 0.05) (S. Figs 3 and 4). The results suggest that the effect of time of day on infection was not strain specific, implying that human chlamydia infection is under the effect of time of day of infection.

### Effect of time of day of infection on chlamydia infectivity and pathogenesis in older mice

**Figure 6:**
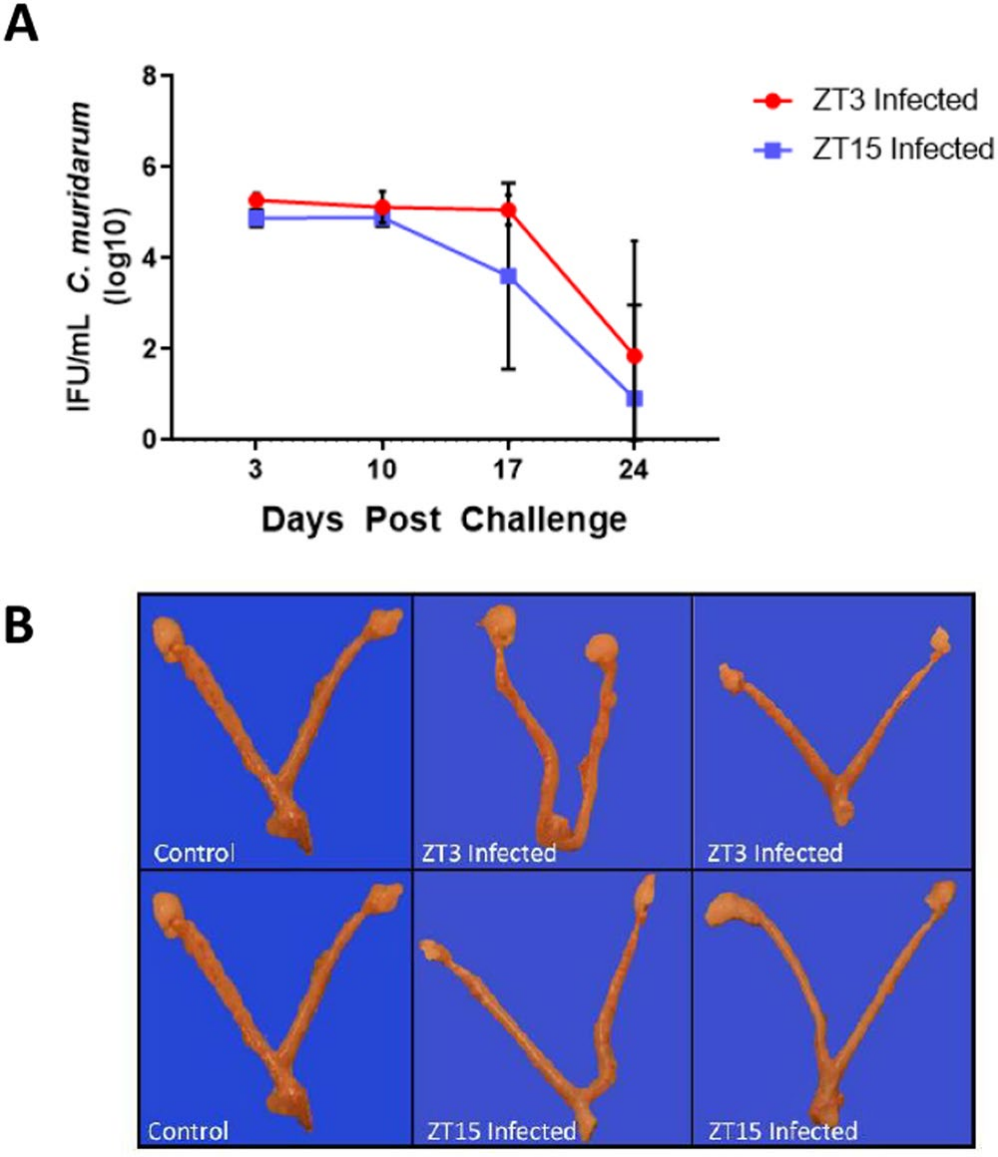
15 weeks old Female mice (n = 7 per group) were housed under LD conditions and infected with *C. muridarum* at either ZT3 or ZT15. Experiment was repeated once. (**A)** There was no significant difference in bacterial burden between mice infected at ZT3 or ZT15. (**B)** There was no difference in gross pathology and histopathological changes when comparing mice infected at either ZT3 or ZT15.

It has been reported that as mice age, the duration of their chlamydia infection is shorter^50^ which mimics the lower incidence of genital chlamydia infection in older human beings^51^. To test the effect of time of day of chlamydia infection on the intensity of infection and development of lesions in older mice, 15-week-old female mice housed under LD conditions were infected and reinfected with *C. muridarum* at either ZT3 or ZT15. The results showed that there was no significant difference in infectivity between ZT3 and ZT15 infected older mice with the duration of the infection being shorter compared to the younger mice (Fig. 6A). Note that the infection was cleared by day 24 in most of the mice, compared to the younger mice that cleared their infection from day 27. In addition, the incidence and severity of significant gross pathology and histopathological changes were greatly diminished when comparing mice infected in the early rest and early active periods (Fig. 6B). The results indicated that the effect of time of day of infection is not apparent in older mice, thus implying that age is an important factor in determining the effect of time of day of infection.

## Discussion

Genital chlamydia infection, a disease caused by infection with *C. trachomatis*, is the most frequently reported bacterial STD in the United States^1,2^. We still do not fully understand why some women are more likely to develop an asymptomatic infection, have severe infection leading to PID and tubal factor infertility, or after exposure to *C. trachomatis* stay uninfected^3,6,7^. We hypothesize that some of these differences amongst individuals infected with *C. trachomatis* might be due to the time of day they were exposed to it.

Our results revealed that under normal LD conditions, mice infected at the early active period shed less bacteria and cleared the infection faster compared to mice infected during the early rest period. The results from our study is corroborated by other studies conducted with pathogens such as herpes simplex virus I and IV, influenza virus and Salmonella, which revealed that mice infected during the active period (at night) had significantly less pathogen burdens compared to the mice that were infected during the early rest period (day)^18,19^. Also, under LD conditions, mice infected with *C. muridarum* during the early active period showed reduced gross lesions with less severe uterine inflammation and dilation compared to mice infected during the early rest period. In addition, development of hydrosalpinx and Bursal cysts was only observed in mice infected during the early rest period. These results corroborate earlier studies, which showed more deaths from endotoxin and pneumococcal infections in nocturnal rodents infected during the late rest period^16,17^. The results reveal that mice can resist genital *C. muridarum* infection when they are infected in the early active period than during the early rest period, indicating a direct linkage between the lesions observed after infection with the time of day of infection. The reason for this difference in pathology could be due to the immune response generated by the chlamydia at the time of infection.

During chlamydia infection TLRs found on immune cells particularly TLR2, TLR4 and TLR9 which are important in recognizing and sensing chlamydia, are highly expressed in the female genital tract where they mediate immune cell response to chlamydia^52–54^. TLR4, TLR5 and TLR9 have been reported to show daily variation in response to their antigens^29,55,56^. We analysed the host immune responses to the infection by measuring the cytokine/chemokine response in the genital washes and chlamydia-specific antibody production. We observed differences in immune response based on the time of day that the mice were infected. For instance, mice infected during the early rest period had significantly higher levels of CXCL-1 in the first week of infection compared to mice infected during the early active period. That implies that more neutrophils were called early to the site of infection, and it has been reported that increased neutrophils are associated with increased pathology^57^, with the depletion of neutrophils associated with reduction in genital tract immunopathology^58^. In addition, neutrophils have been shown to be recruited in the early rest period during an inflammatory response and this rhythmic recruitment was associated with epithelial cells producing chemokines^29^. It should be noted that the epithelial cells in the female genital tract are referred to as sentinels of immune protection recognizing pathogens and signalling underlying immune cells^59,60^. Here we show that clusters of neutrophils in infected mice persisted in the genital tract tissue all through the infection. Mice infected during the early rest period had higher proinflammatory cytokine secretion compared to mice infected during the early active period and significantly higher concentrations of IL-10, IFN-γ, and IL-1β. We do not know the exact cells responsible for producing the cytokines, but this could be attributed to the innate cells recruited early to the site of infection; mitigated by the expression of TLRs in the genital tract; such as the natural killer cells (NK) cells, monocytes, macrophages, dendritic cells and the epithelial cells that are already part of the physical barrier providing protection in the genital tract^52,56,58,59,61–66^. IFN-γ and IL-1β are important in clearing and reducing the bacterial burden, by helping to elicit a proper Th1 response^23–25^. IFN-γ is produced early by NK and other immune cells, and may be modulating DC function towards Th1 activation^67^. However, very high amounts of IL-1β and other pro-inflammatory cytokines have been associated with detrimental pathologies observed during chlamydia infection^57,68^. There is often a high concentration of anti-inflammatory cytokine IL-10 to counter the effects of high concentrations of proinflammatory cytokines, which we observed in the mice infected during the early rest period^69^. In general, a high inflammatory response is beneficial in clearing an infection, but prolonged inflammation can lead to tissue damage^57^. We believe that the differences observed in the lesions of mice infected during the early rest or active periods are associated with cytokine response.

The presence of anti-chlamydia IgG, IgG2C, and IgA are indicative of a current or chronic chlamydia infection and to a certain extent protection from chlamydia infection^70^. Under normal light conditions serum levels of IgG were significantly higher in mice infected during the early active period in the first week of infection. However, in the second and third weeks of infection, mice infected during the early rest period had significantly higher IgG. It is possible that having an infection during the early active period elicits IgG quickly, which leads to the faster clearance of infection compared to mice infected during the early rest period. In the first, second and third weeks of infection, mice infected during the early rest period had significantly higher IgG2C than mice infected during the early active period. It should be noted that the IFN-γ produced during the early stages of chlamydia infection has been associated with the production of the Th1 cytophilic antibody IgG2a (in BL6 mice; IgG2c)^67^. Here we show a strong correlation between IFN-γ and IgG2c secretion in mice infected at ZT3. This Th1 associated antibody might be important in process of reducing the high bacteria burden in mice infected during the early rest period. There was no significant difference in the expression of chlamydia specific serum IgA between mice infected during the early rest or active periods. However, we noticed a dramatic increase in mucosal IgA in mice infected during the early active period, four weeks after the infection, this might be associated with chlamydia clearance and longer protective immunity.

To buttress the effect of time of day of infection on infectivity and pathology, we decided to infect mice twice with *C. muridarum*. Following reinfection, a pattern like we had observed after a single infection, with mice infected during the early rest period having more infectivity and pathology than mice infected during the early active period. Mice are nocturnal and are more active at night^71^, and we infected about 3 hours into the mice active period. Based on the infectivity and pathology data, mice infected with *C. muridarum* during the active period results in less severe lesions and disease outcomes compared to having an infection during their early rest period. However, in humans the reverse might be the case since we are diurnal organisms. This suggests that in humans, infection with *C. trachomatis* during the day might result in less severe infection and reduced complications when compared to night infection. This would have ramifications in the public health status of individuals especially those who are more liable to be active at night such as prostitutes and maybe even shift workers.

Women who are infected with *C. trachomatis* have a greater chance of becoming infertile^6,7^. Reinfection leads to increased pathology and infertility, here we used mice that were reinfected with *C. muridarum* to determine the effect of time of day of chlamydia infection on fertility outcomes. There was no significant difference in the fertility rate between the groups; however, there was a significant difference in the number of pups, with mice infected during the early active period having a significantly greater number of pups than mice infected during the early rest period. This is intriguing as there appears to be a direct relationship between time of infection and number of pups; fertility rate. We do not know if this relationship exists in women of childbearing age infected with *C. trachomatis*.

Our results suggest that the effect of time of day of infection was not strain specific or age dependent. Like *C. muridarum* infected mice, mice infected with human chlamydia serovar, *LGV*, had higher infectivity if the infection was during the early rest period compared to infection during the early active period. They were also less fertile if they were infected during the early rest period. In addition, we investigated the effect of age on this phenomenon, since it is known that older mice have a shorter duration of genital chlamydia infection^50^. Our results showed that older mice in general cleared their infections faster, however, the trend was similar with mice infected during the early active period having lower bacterial burden than mice infected during the early rest period.

Time of day of chlamydia infection appears to have an influence on its pathogenesis and disease progression. The time of day that an individual becomes infected with *C. trachomatis* could explain why there are differences in susceptibility and severity among individuals. It may also predict why some individuals are asymptomatic while others are not. This can be linked to the rhythmic changes in the production of chemokines/cytokines and antibodies. The numbers of immune cells found at the site of an infection have been shown to be regulated by the SCN through humoral or neuronal coordination^33,47–49^. However, we do not know if the production of the immune effectors observed in our study, is controlled by the clock within the immune cells or the oscillators associated with the clocks. Mechanistic studies should be carried out to understand the circadian control of immune cells in chlamydia infection and pathogenesis. Female sex hormones have been postulated to control immune response to chlamydia infection^52^, further work on understanding how this phenomenon happens at different times of the day would be important in elucidating chlamydial pathogenesis. Overall, findings from this study have implications for shift workers and individuals who do not have a normal sleep schedule. The circadian clocks of shift workers are disrupted causing them to potentially become more susceptible to diseases in general. These individuals, especially women, could be more at risk for developing a more severe genital chlamydia infection leading to infertility. This study provides an opportunity from which to start threading together the numerous innovative developments in the circadian field into elucidating the mechanisms underlying chlamydial pathogenesis.

## Materials and Methods

### Animal protocol approval statement

This study was carried out in strict adherence with the recommendations in the Guide for the Care and Use of Laboratory Animals of the National Institutes of Health. The Institutional Animal Care and Use Committee (IACUC) of Morehouse School of Medicine approved the study protocol (Protocol Number: 16–24).

### Animals

Female C57BL/6J mice (Jackson Laboratory, Bar Harbor, MA) received at six weeks and 15 weeks old were housed under normal light: dark cycle conditions of 12 hours light: 12 hours dark (LD). The light intensity during the light phase in the room is 906 Lux.

### *Chlamydia muridarum* stock

*Chlamydia muridarum niggs* (*C. muridarum*) and *Chlamydia trachomatis* (*C. trachomatis*), *Lymphogranuloma venereum* (LGV) stocks (Centers for Disease Control, Atlanta, GA) were diluted in sterile Sucrose Phosphate Glutamate (SPG) transport media to a final concentration of 1 × 10^5^ Infectious Units (IFUs).

### C. muridarum infectivity assay

All mice were subcutaneously injected between 10:00 am and 12:00 noon with 2.5 mg/mouse Depo Provera, medroxyprogesterone acetate (Pfizer, New York, NY) in sterile Phosphate Buffer Saline (PBS) to synchronize the estrous cycle. Mice were intravaginally infected seven days later, with 1 × 10^5^ *C. muridarum* at 10:00 am (ZT3, early rest period) or 10:00 pm (ZT15, early active period), which can be interpreted as three hours after the lights were turned on or off in the room (7:00 am lights on, 7:00 pm lights off). The mice were anesthetized using isoflurane during process of infection. For a repeat infection or reinfection, mice were infected 4 weeks after following the same process mentioned above. Infected and reinfected mice were swabbed every three days for 27 days and the bacteria was isolated and cultured to track the progression and clearance of the infection.

### Gross pathology

Mice were infected and reinfected intravaginally with *C. muridarum* (1 × 10^5^ IFU per mouse). All mice were sacrificed four weeks after infection, between 10:00 am and 12:00 noon and the entire genital tract from the vagina to the ovary was collected and fixed in neutral-buffered, 10% formalin. Euthanasia was carried out using carbon dioxide and cervical dislocation. Gross pathology was performed by counting and observing the numbers of paraovarian cysts and tubal dilations in the genital tract of chlamydia infected mice. Note that the collection was synchronised to make sure that all samples were collected within the same period.

### Histopathology

Histopathology of the genital tract associated with chlamydia infection was investigated. Mice were infected and reinfected intravaginally with *C. muridarum* (1 × 10^5^ IFU per mouse). All mice were sacrificed four weeks after infection between 10:00 am and 12:00 noon, and the entire genital tract from the vagina to the ovary was collected and fixed in neutral-buffered, 10% formalin solution for less than 1 week. Tissues were routinely processed, embedded in paraffin, cut approximately into 5 μm sections, and stained with hematoxylin and eosin (H and E). Histopathological exam consisted of evaluation of the ovaries, oviducts, and uteri for the incidence (presence or absence), severity, and distribution of inflammation, necrosis, and hyperplasia. Histopathologic severity scores were assigned as grades 0 (no significant histopathological alterations); 1 (minimal); 2 (mild); 3 (moderate); or 4 (severe) based on an increasing extent and/or complexity of change, unless otherwise specified. Lesion distribution was recorded as focal, multifocal, or diffuse, with distribution scores of 1, 2, or 3, respectively. A total severity-and-distribution group score was calculated by adding individual distribution and severity scores. Because the group sizes were uneven, an average severity-and-distribution group score was calculated by dividing the total severity-and-distribution score by the numbers of animals in the group.

### Cytokine and chemokine assay

Vaginal lavages were collected every week between 10:00 am and 12:00 noon throughout the duration of the infection and the amount of cytokines (CXCL1, TNF-α, IL-10, IL-1β and IFN-γ) produced was determined using the R&D Systems Magnetic Luminex Assay, Mouse Premixed Multi-Analyte Kit (R&D, Hercules, CA) in accordance with the manufacturer’s protocol. The concentration of cytokine in each sample was obtained by extrapolation from a standard calibration curve. The mean and SD of all replicate cultures were calculated.

### Enzyme linked immunosorbent assay

Blood samples were collected every week between 10:00 am and 12:00 noon throughout the duration of the infection and serum was collected by centrifuging the blood sample at 2500 rpm for 2 minutes. Determination of concentrations of chlamydia-specific antibody isotypes (IgG, IgG2C, and IgA) in vaginal lavage and serum was measured by a standard ELISA procedure described previously^72^. UV inactivated. *C. muridarum* elementary bodies (10 µg/ml) in 50 µl of PBS was used to coat 96 – well plates (Nunc Life Technologies, Rochester, NY) overnight at 4 °C. Plates were blocked with 1% bovine serum albumin containing 5% goat serum in PBS – tween. Vaginal lavage (50 µl) in a twofold serial dilution were added to each well. Horseradish peroxidase – conjugated goat – anti-mouse IgG and IgA isotypes (50 µl) (Southern Biotechnology Associates, Inc., Birmingham, AL) were added to each plate and incubated for one hour and developed with 2,2′-azino-bis (3-ethylbenzthiazoline-6-sulfonic acid) (ABTS). The optical density was measured at 490 nm on a microplate reader and the results were generated with a standard curve. Data sets are displayed using the corresponding absorbance values as mean concentrations (ng/ml) ± SD and represent the mean values from triplicate experiments.

### Fertility assay

Three weeks after reinfection, female uninfected control, mice infected at ZT3 and ZT15 were placed in cages with proven fertile male C57Bl/6J mice (Jackson Laboratory, Bar Harbor, MA), at either three females or two females to one male mouse. The female mice were weighed every three days after one week until they have gained approximately 10 grams to confirm pregnancy. Once pregnancy has been confirmed, mice were sacrificed and dissected to determine the number of pups^73,74^.

### Statistical analysis

Nonparametric one-way analysis of variance (ANOVA) was used to analyse the statistical differences in immune response and fertility rate between the treatment groups. Two-way ANOVA was used to determine the difference in infectivity between the treatment groups. In addition, we also did a post hoc test after the one way and two-way ANOVA, to determine the actual statistical relationship between the treatment groups. Chi-square test was used to determine the difference in pregnancy rate between the different treatment groups. Statistical significance was determined at P < 0.05. GraphPad Prism (La Jolla, CA) is the statistical package that was used for analysing the data.

## Supplementary information

Supplementary data

## Data Availability

All data and results have been added to this manuscript and the Supplementary Material section.
