# Supplementary material for: Effect of Time of Day of Infection on Chlamydia Infectivity and Pathogenesis
Source: Sci Rep. 2019 Aug 6;9:11405. doi: 10.1038/s41598-019-47878-y (PMC6684580; doi:10.1038/s41598-019-47878-y)
Supplement: Supplementary file 1 — Supplementary data [file 41598_2019_47878_MOESM1_ESM.pdf]

Effect of Time of Day of Infection on Chlamydia Infectivity and Pathogenesis

Stephanie Lundy[1], Tarek Ahmad[1], Tankya Simoneaux[1], Ifeyinwa Benyeogor[1], YeMaya Robinson[1,6], Zenas George[2], Debra Ellerson[2], Ward Kirlin[3], Tolulope Omosun[4], Francis O. Eko[1], Carolyn M. Black[2], Uriel Blas-Machado[5], Jason P. DeBruyne[3], Joseph U. Igietseme[1, 2], Qing He[1, 2] and Yusuf Omosun[*1, 2].

[1]Department of Microbiology, Biochemistry & Immunology, Morehouse School of Medicine, Atlanta, GA 30310, USA; [2] Molecular Pathogenesis Laboratory, Centers for Disease Control & Prevention (CDC), Atlanta, GA 30333, USA; [3]Department of Pharmacology and Toxicology, Morehouse School of Medicine, Atlanta, GA 30310, USA; [4] Department of Physical Sciences, Georgia State University, Covington, GA 30014; [5] Department of Pathology and Athens Veterinary Diagnostic Laboratory, University of Georgia, College of Veterinary Medicine, Athens, GA 30602, USA. [6] Spelman College, Atlanta, GA 30314, USA

Short Title: Time of day determines Chlamydia infection

Supplementary Figures

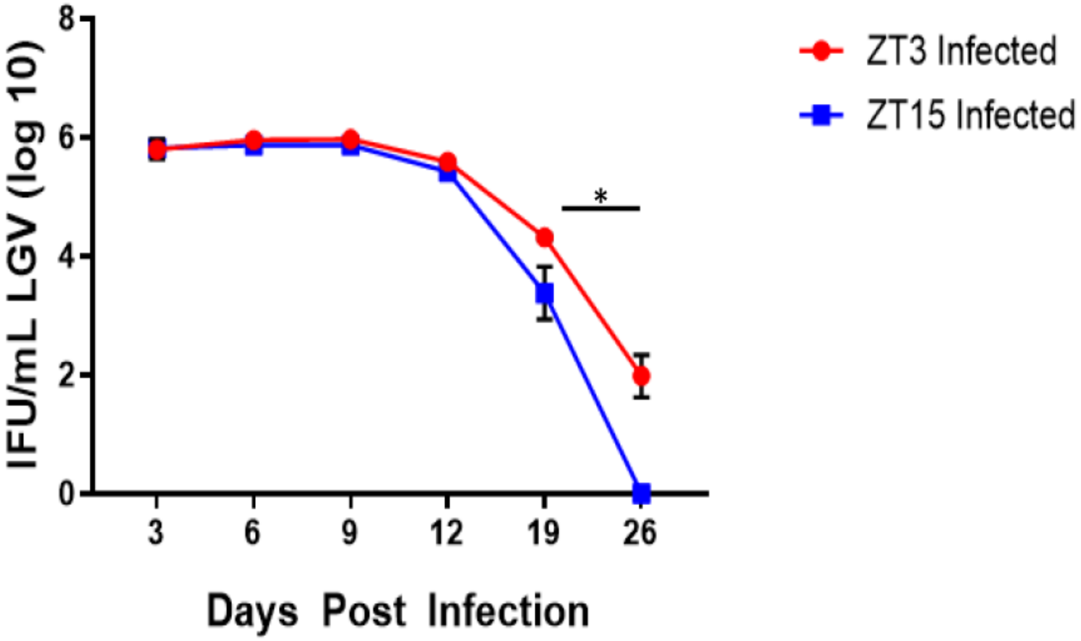

S. Fig. 1: Effect of time of day of infection on mice infected with LGV. Female mice (n=6 per group) housed under LD conditions and were infected with LGV at ZT3 or ZT15. Experiment was repeated once. Mice infected at ZT3 had significantly higher infectivity compared to mice infected at ZT15 from days 19 and 26 (****p < 0.0001) (two-way ANOVA).

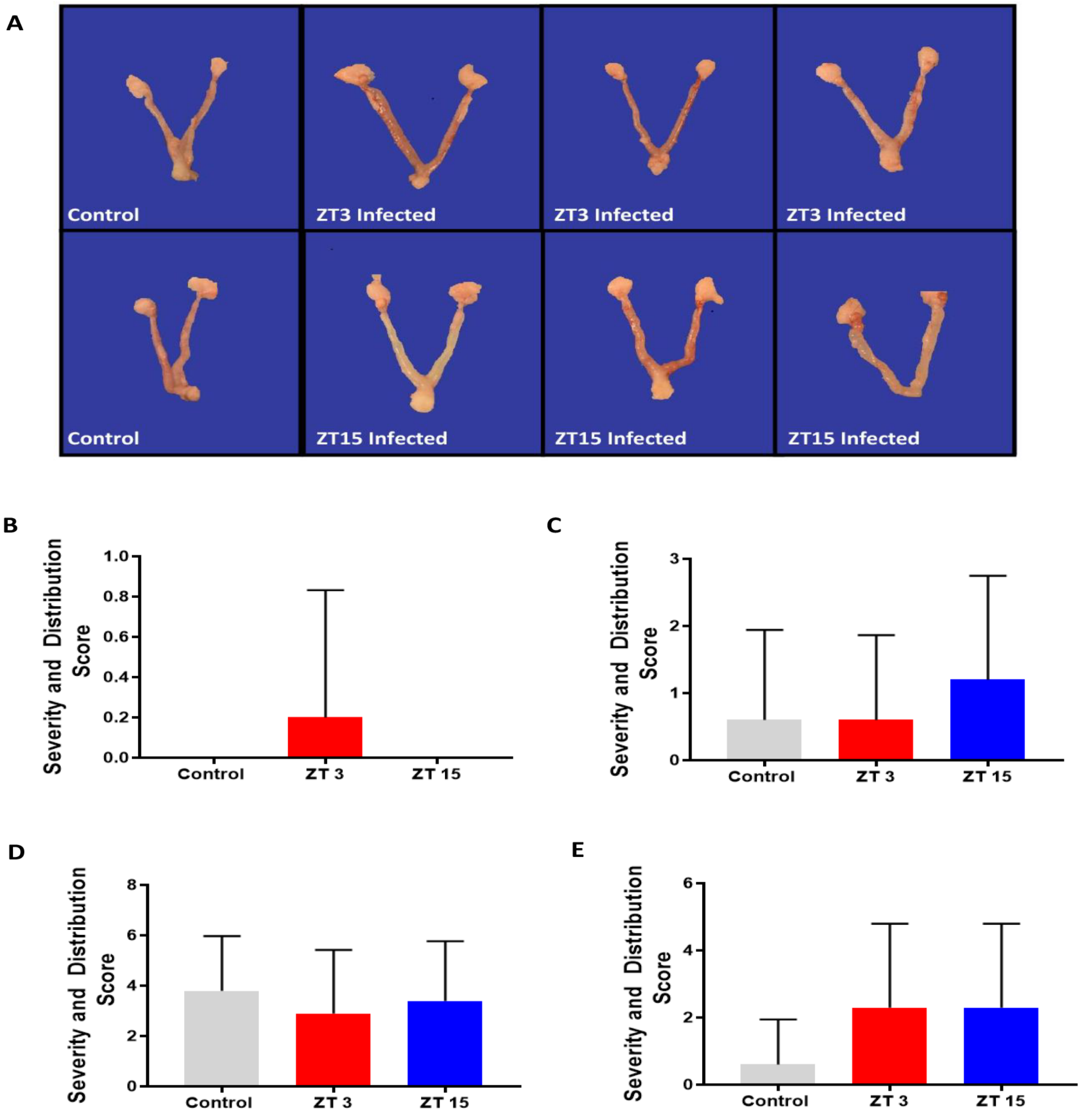

S. Fig. 2: Gross pathology and histopathology in female mice housed under LD conditions. Mice (n=12 per group) were infected with *LGV* at either ZT3 or ZT15. A) Gross pathology of genital tract. The scores for the genital tract histopathology were presented as follows, B) Ovary Inflammation. C) Uterus Inflammation. D) Uterus Myxedema. E) Uterus Hyperplasia. The ovaries of mice infected at ZT3 had more inflammation than mice infected at ZT15, while in the uterus there was no significant difference between the two groups. The histopathology scores were analyzed using a one-way ANOVA and post hoc test.

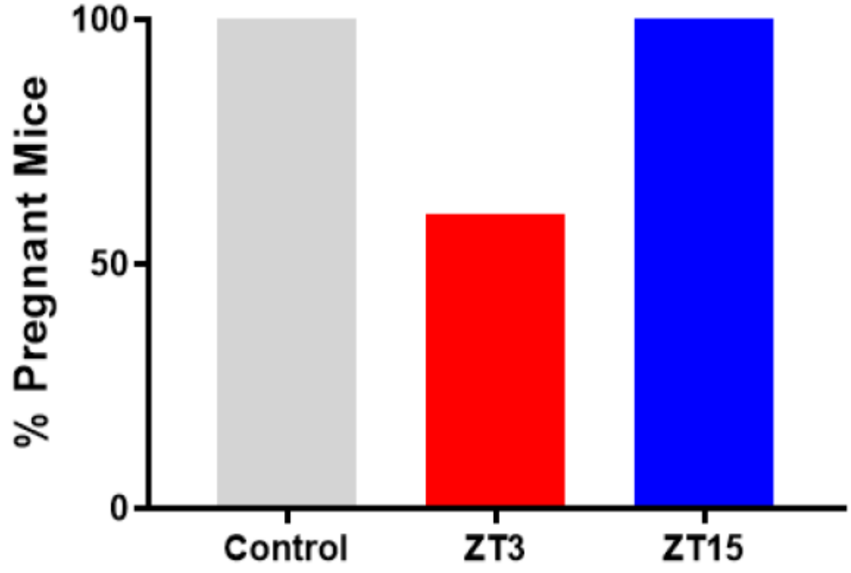

S. Fig. 3: Effect of time of day of infection on mice infected with LGV. Female mice (n=6 per group) housed under LD conditions and were infected with LGV at ZT3 or ZT15. Experiment was repeated once. No significant difference in pregnancy rate between the mice infected at ZT3 or ZT15, (Chi-square test).

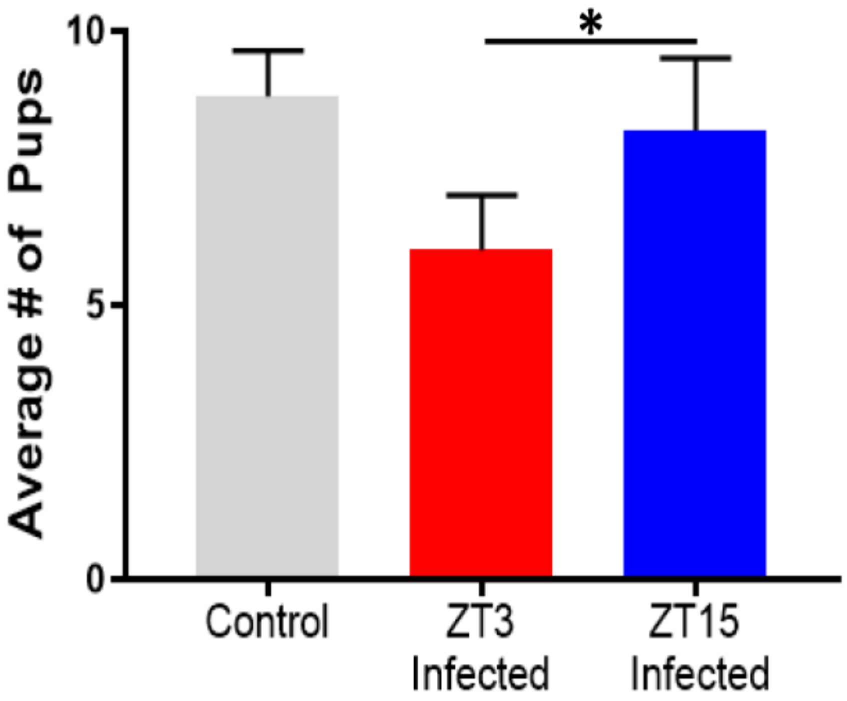

S. Fig. 4: Effect of time of day of infection on mice infected with LGV. **A)** Female mice (n=6 per group) housed under LD conditions and were infected with LGV at ZT3 or ZT15. Experiment was repeated once. Mice infected at ZT15 had a significantly greater number of pups compared to mice infected at ZT3 (*p<0.05) (one-way ANOVA).
